# Supplementary material for: ZIKV infection induces robust Th1-like Tfh cell and long-term protective antibody responses in immunocompetent mice
Source: Nat Commun. 2019 Aug 27;10:3859. doi: 10.1038/s41467-019-11754-0 (PMC6712032; doi:10.1038/s41467-019-11754-0)
Supplement: Supplementary file 1 — Supplementary Information [file 41467_2019_11754_MOESM1_ESM.pdf]

Supplementary information

ZIKV infection induces robust Th1-like Tfh cell and long-term protective antibody responses in immunocompetent mice

Huabin Liang[1,2#], Jinyi Tang[1,2#], Zhihua Liu[1,2#], Yuanhua Liu[1], Yuanyuan Huang[1,2], Yongfen Xu[1], Pei Hao[1], Zhinan Yin[3], Jin Zhong[1], Lilin Ye[4], Xia Jin[1,2*], Haikun Wang[1,2*]

1. CAS Key Laboratory of Molecular Virology and Immunology, Institut Pasteur of Shanghai, Chinese Academy of Sciences, Shanghai, China
2. University of Chinese Academy of Sciences, Beijing, China.
3. The First Affiliated Hospital, Biomedical Translational Research Institute, Guangdong Province Key Laboratory of Molecular Immunology and Antibody Engineering, Jinan University, Guangzhou, China.
4. Institute of Immunology, Third Military Medical University, Chongqing, China.

Table 1. Related to materials and methods. Antibodies used in this study

Antibody	Dilution	Fluorophor	Clone No.	Cat No.	Provider
**CD4**	1:200	BV421	GK1.5	100437	Thermo Fisher Scientific
**CD4**	1:200	PerCP- eFluor 710	GK1.5	46-0041-80	eBioscience
**CD4**	1:200	APC	GK1.5	17-0041-83	eBioscience
**CD4**	1:200	APC-eFluor 780	GK1.5	47-0041-80	eBioscience
**CD8a**	1:200	PerCP	53-6.7	100732	Thermo Fisher Scientific
**CD8a**	1:100	Pacific Blue	53-6.7	558106	BD Biosciences
**CD44**	1:200	FITC	IM7	11-0441-82	eBioscience
**CD44**	1:200	APC	IM7	17-0441-82	eBioscience
**CD44**	1:200	BV605	IM7	103047	Thermo Fisher Scientific
**CD45.2**	1:200	Percp- cyanine5.5	104	45-0454-82	eBioscience
**CD45.2**	1:200	FITC	104	109806	BiogLend
**CD45.2**	1:200	PE- cyanine7	104	25-045482	eBioscience
**CD62L**	1:200	APC	MEL-14	17-0621-82	eBioscience
**CD62L**	1:200	APC- eFluor 780	MEL-14	47-0621-82	eBioscience
**CXCR3**	1:200	APC	CXCR3-173	126512	Thermo Fisher Scientific
**CXCR5**	1:50	PE	SPRCL5	12-7185-82	eBioscience
**CXCR5**	1:200	biotin	SPRCL5	13-7185-82	eBioscience
**PD-1**	1:200	PE- cyanine7	29F.1A12	135216	Thermo Fisher Scientific
**GL-7**	1:200	eFluor 450	GL-7(GL7)	48-5902-82	eBioscience
**GL-7**	1:200	Alexa Fluor 647	GL7	561529	BD Biosciences
**PNA**	1:200	FITC	Lack	L7381	Sigma
**FAS**	1:200	PE- cyanine7	Jo2	557653	BD Biosciences
**B220**	1:200	APC	RA3-6B2	17-0452-81	eBioscience
**B220**	1:200	BV605	RA3-6B2	103243	Thermo Fisher Scientific
**IgD**	1:200	eFluor 450	11-26c(11-26)	48-5993-82	eBioscience
**IgM**	1:200	APC- eFluor 780	II/41	47-5790-82	eBioscience
**IgG1**	1:200	Percp	RMG1-1	406611	Thermo Fisher Scientific
**IgG2b**	1:200	biotin	RMG2b-1	406704	Thermo Fisher Scientific
**IgG2c**	1:200	FITC	Lack	1079-02	SouthernBiotech
**IFN-γ**	1:200	PerCP- cyanine5.5	XMG1.2	45-7311-82	eBioscience
**IL-4**	1:100	BV421	11B11	504120	Thermo Fisher Scientific
**IL-4**	1:200	PE- cyanine7	11B11	25-7041-82	eBioscience
**IL17a**	1:200	Alexa Fluor 700	TC11-18H10	560820	BD Biosciences
**IL17a**	1:200	APC	eBio17B7	17-7177-81	eBioscience
**BCL6**	1:50	PE	7D1	358504	Thermo Fisher Scientific
**ICOS**	1:200	Alexa Fluor 647	7E.17G9	563469	BD Biosciences
**CD40L**	1:100	APC	MR1	17-1541-82	eBioscience
**T-BET**	1:100	Alexa Fluor 660	eBio4B10	50-5825-82	eBioscience
**EOMES**	1:100	PerCP- eFluor 710	Dan11mag	46-4875-82	eBioscience
**LY6C**	1:200	PE	RB6-8C5	553128	BD Biosciences
**SLAM**	1:200	BV785	TC15- 12F12.2	115937	Thermo Fisher Scientific
**Streptavidin**	1:100	BV421	Lack	563259	BD Biosciences
**Streptavidin**	1:200	PE	Lack	554061	BD Biosciences
**IL-21**	1:50	IL-21R Fc Chimera	Lack	596-MR	R&D Systems
**Anti-Human IgG**	1:500	Alexa Fluor 647	Lack	109-606-088	Jackson
**CD8a**	1:500	biotin	53-6.7	553029	BD Biosciences
**TER-119**	1:500	biotin	Lack	553672	BD Biosciences
**B220**	1:500	biotin	RA3-6B2	553086	BD Biosciences
**NKG2D**	1:200	APC	CX5	17-5882-81	Thermo Fisher Scientific
**NK1.1**	1:200	PerCP- cyanine5.5	PK136	561111	BD Biosciences

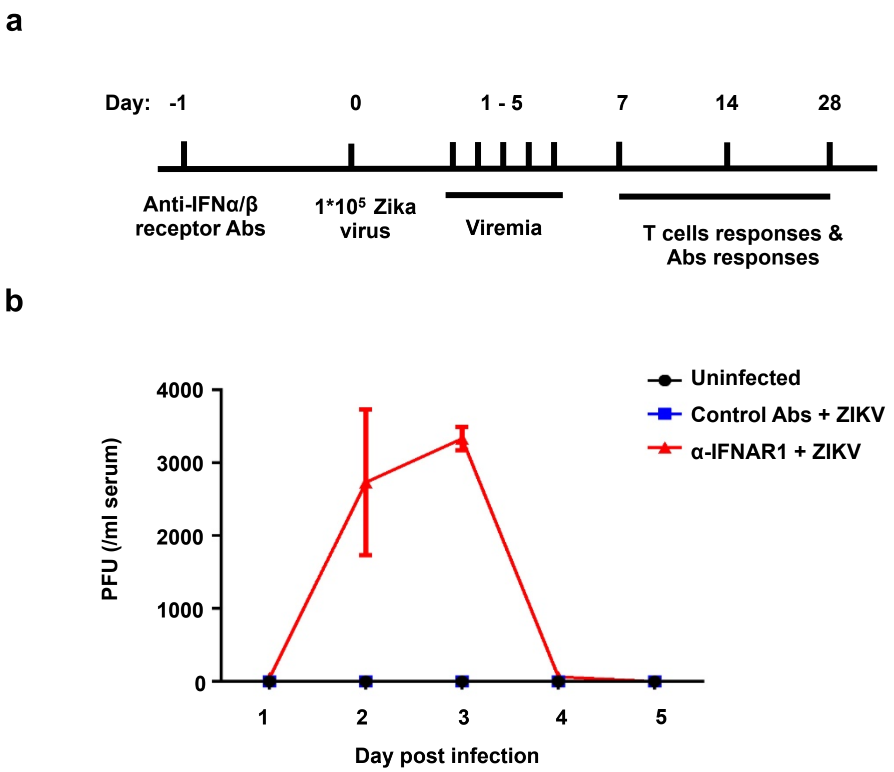

Supplementary Figure 1. Establishment of a murine model of ZIKV infection.

(a) Experimental strategy for infection, viremia detection and immune response tests at different day post infection (dpi). **(b)** Sera from uninfected mice, ZIKV infected mice administered with control antibody or anti-IFNα/β receptor antibody (anti-IFNAR1) were collected from 1 to 5 dpi. Viremia levels were quantified by plaque assay on Vero cells (n=3 for each group). The summary data were presented as mean ± SEM. Source data are provided as a Source Data file.

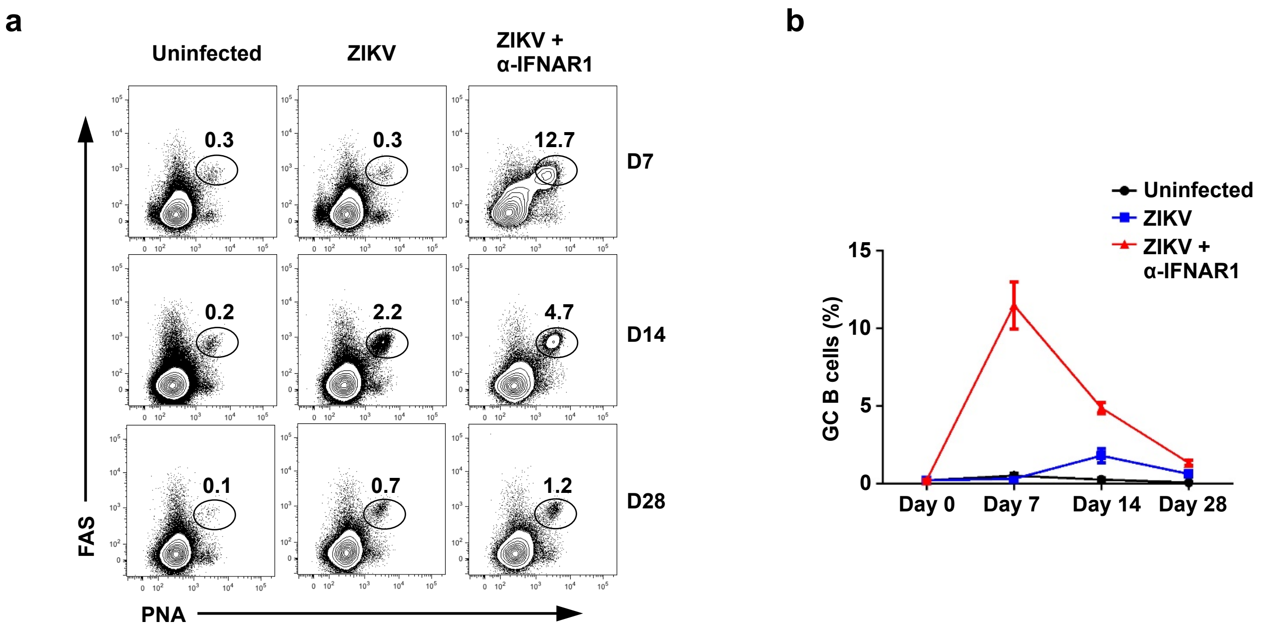

Supplementary Figure 2. Immune-modulated ZIKV infection induces strong germinal center B cell responses. **(a-b)** BALB/c mice were administered with PBS control, ZIKV only, or ZIKV+anti-IFNAR1 antibody, and splenocytes were harvested on 7, 14 and 28 dpi for staining with different surface markers (n=3 for each group). **(a)** Representative flow cytometry plots of Fas^+PNA^+ germinal center (GC) B cell responses gated from $B220^+$ B cells. **(b)** Summary of the kinetic changes of GC B cells; The summary data were presented as mean ± SEM. Source data are provided as a Source Data file.

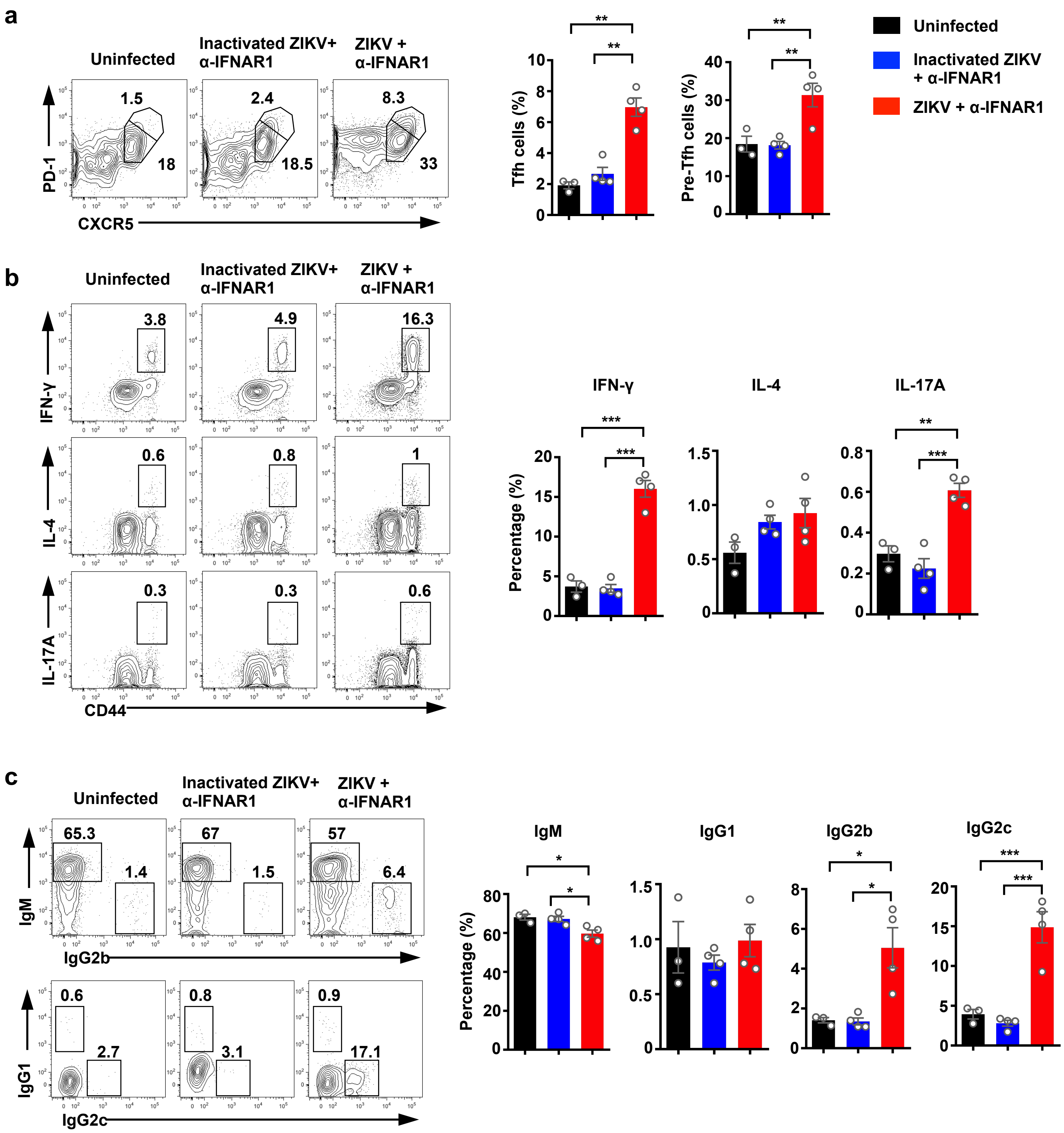

Supplementary Figure 3. Replication of ZIKV is required for robust Tfh cell and B cell responses.

C57BL/6 mice were administered with PBS, inactivated ZIKV or live ZIKV with anti-IFNAR1 antibody pre-treatment. Splenocytes were collected on 7 dpi for Tfh (n=3 for PBS group, and n=4 for inactivated ZIKA and live ZIKV group); splenocytes were collected on 14 dpi for other helper T cell subsets cytokines and antibody response staining. Representative flow cytometry plots of **(a)** Tfh and pre-Tfh cells, **(b)** IFN-γ^{+} cells (Th1), IL-4^{+} cells (Th2) and IL-17A^{+} (Th17) cells in CD4^{+} T cells, **(c)** IgM^{+}, IgG1^{+}, IgG2b^{+} and IgG2c^{+} B cells, with bar graphs summarized the percentages. The summary data were presented as mean ± SEM. Statistical differences were determined by Student's t test and p values were indicated by * (p<0.05), or ** (p<0.01), or *** (p<0.001). Source data are provided as a Source Data file.

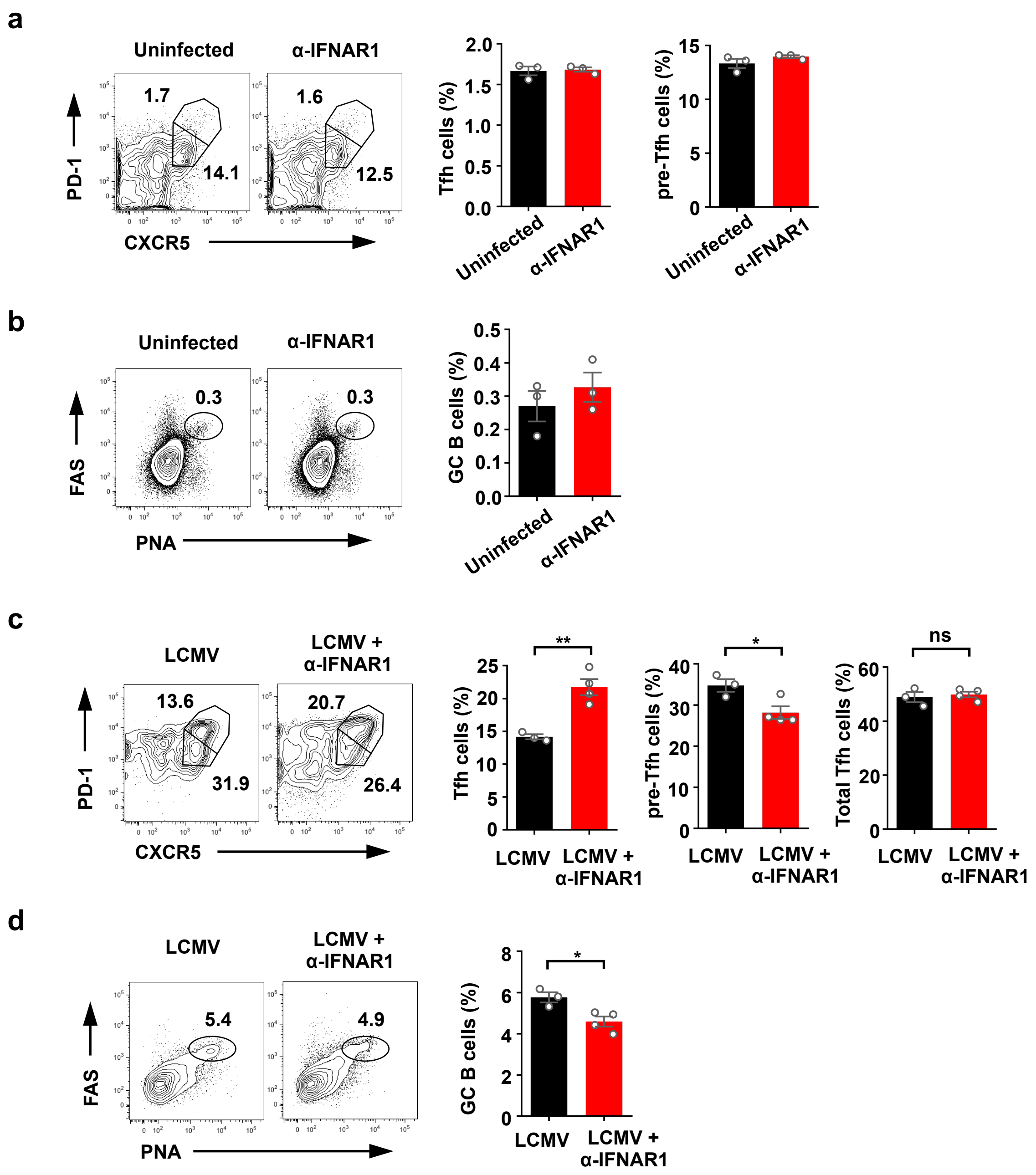

Supplementary Figure 4. The effect of anti-IFNAR1 antibody on Tfh and GC B cell responses in uninfected- or LCMV-infected mice.

(a-b) C57BL/6 mice were administered with or without anti-IFNAR1 antibody, spleens were harvested on 7 dpi for assessment of **(a)** Tfh and pre-Tfh cells, and **(b)** GC B cells (n=3 for each group). **(c-d)** C57BL/6 mice were infected by LCMV with or without anti-IFNAR1 antibody administered at 1 day prior to infection, spleens were harvested on 7 dpi for measurement of **(c)** Tfh and pre-Tfh cells and **(d)** GC B cells (n=3 for LCMV only group; n=4 for LCMV+ anti-IFNAR1 group). **(a, c)** Representative flow cytometry plots of $CXCR5^{high}PD\text{-}1^{high}$ cells (Tfh) and $CXCR5^{medium}PD\text{-}1^{medium}$ cells (pre-Tfh) in $CD4^{+}CD44^{high}CD62L^{low}$ cells (Left panel); with bar graphs summarized cell percentages (Right panel). **(b, d)** Representative flow cytometry plots of $FAS^{+}GL7^{+}$ cells in $B220^{+}$ B cells (GC B cells) (Left panel); with bar graphs summarized cell percentages (Right panel). The summary data were presented as mean ± SEM. Statistical differences were determined by Student's t test and p values were indicated by ns, not significant (P > 0.05), * (p<0.05), or ** (p<0.01). Source data are provided as a Source Data file.

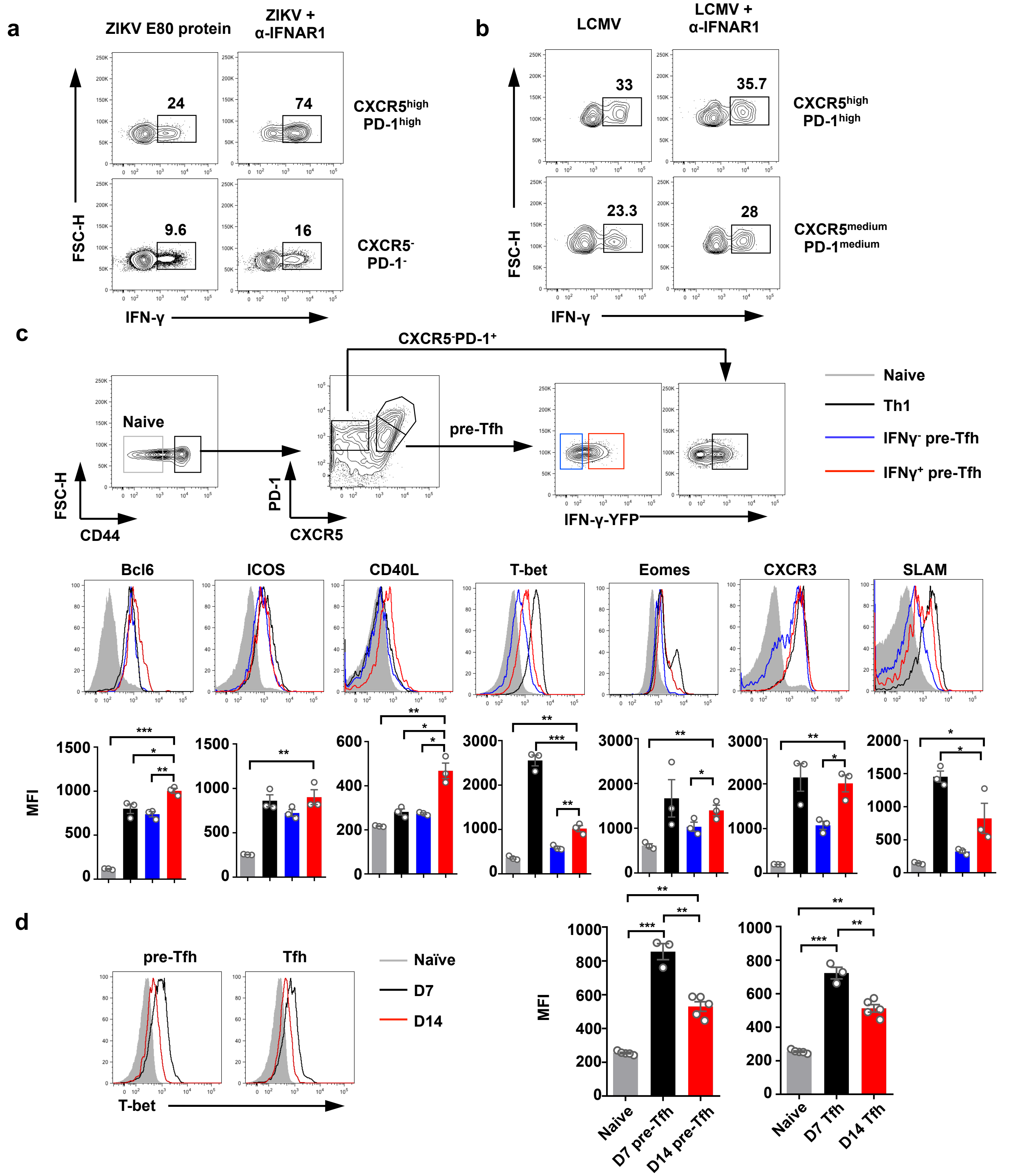

Supplementary Figure 5. Th1-like Tfh and Th1-like pre-Tfh induced specifically by ZIKV infection have characteristic phenotypic profile. **(a)** GFP^{hi}PD-1^{hi} (Tfh) and GFP^{low}PD-1^{low} (non-Tfh) cells were sorted from spleen of CXCR5-GFP reporter mice that were administered with either ZIKV envelope protein+ Alum or ZIKV with anti-IFNAR1 antibody pretreatment. Intracellular staining of IFN-γ were shown as a percentage in sorted cells. **(b)** IFN-γ expressing cells were examined in pre-Tfh cells and the Tfh cells in LCMV only or LCMV+ anti-IFNAR1 group. **(c)** Gating strategy of naïve cells, Th1 cells, IFNγ$^-$ pre-Tfh and IFN-γ$^+$ pre-Tfh cells in IFN-γ–YFP reporter mice. The expression of Bcl6, ICOS, CD40L, T-bet, Eomes, CXCR3 and SLAM in naïve, Th1, IFNγ$^-$ pre-Tfh and IFN-γ$^+$ pre-Tfh cells were presented as histograms. Mean fluorescence intensity (MFI) of the expression of each protein expression was summarized with bar graph (n=3 for each group). **(d)** Expression of T-bet in naïve, Tfh or pre-Tfh cells on 7 or 14 dpi were presented as histograms (Left panel), with bar graph summarized the MFI of T-bet expression in each group (Right panel) (n=3-5 for each group). The summary data were presented as mean ± SEM. Statistical differences were determined by Student's t test and p values were indicated by * ($p<0.05$), or ** ($p<0.01$), or *** ($p<0.001$). Source data are provided as a Source Data file.

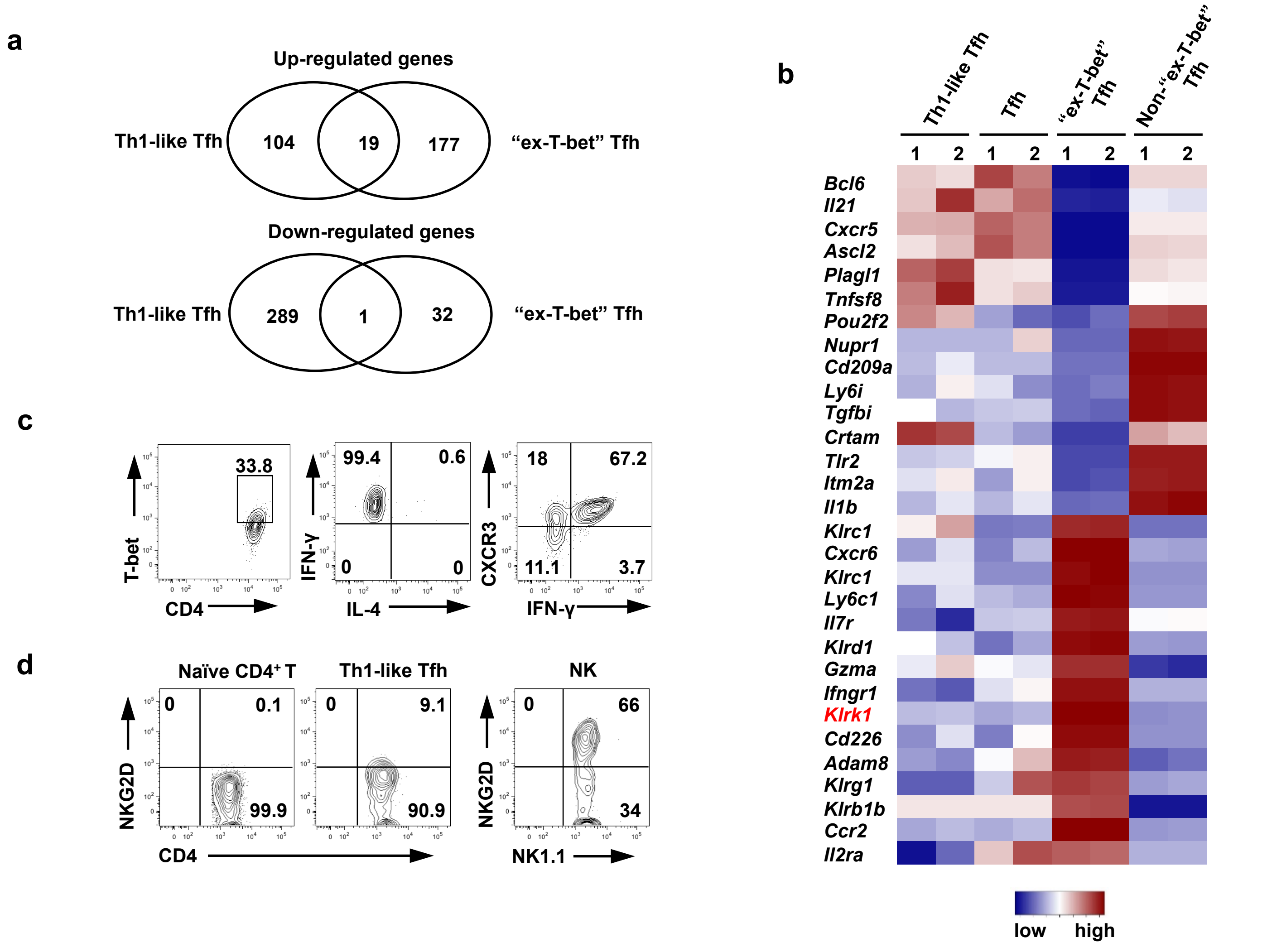

Supplementary Figure 6. Th1-like Tfh cells are distinct from "ex-T-bet" Tfh cells.

(a-b) RNA-seq data set of Th1-like Tfh cells with that the RNA-seq data set of "ex-T-bet" Tfh cells in the recent study (Fang *et al*., JEM, 2018, Nov 5;215(11):2705-2714.) were compared. **(a)** The up-regulated and down-regulated genes in Th1-like Tfh cells and "ex-T-bet" Tfh cells ($p<0.05$, Fc>2). **(b)** Heatmap of Tfh and Th1 related key genes in Th1-like Tfh cells and "ex-T-bet" Tfh cells. **(c)** Representative flow cytometry plots of T-bet, IFN-γ and CXCR3 in Th1-like Tfh cells. **(d)** Representative flow cytometry plots of NKG2D staining in Th1-like Tfh cells, negative control naïve CD4+ T cells, and positive control NK cells.

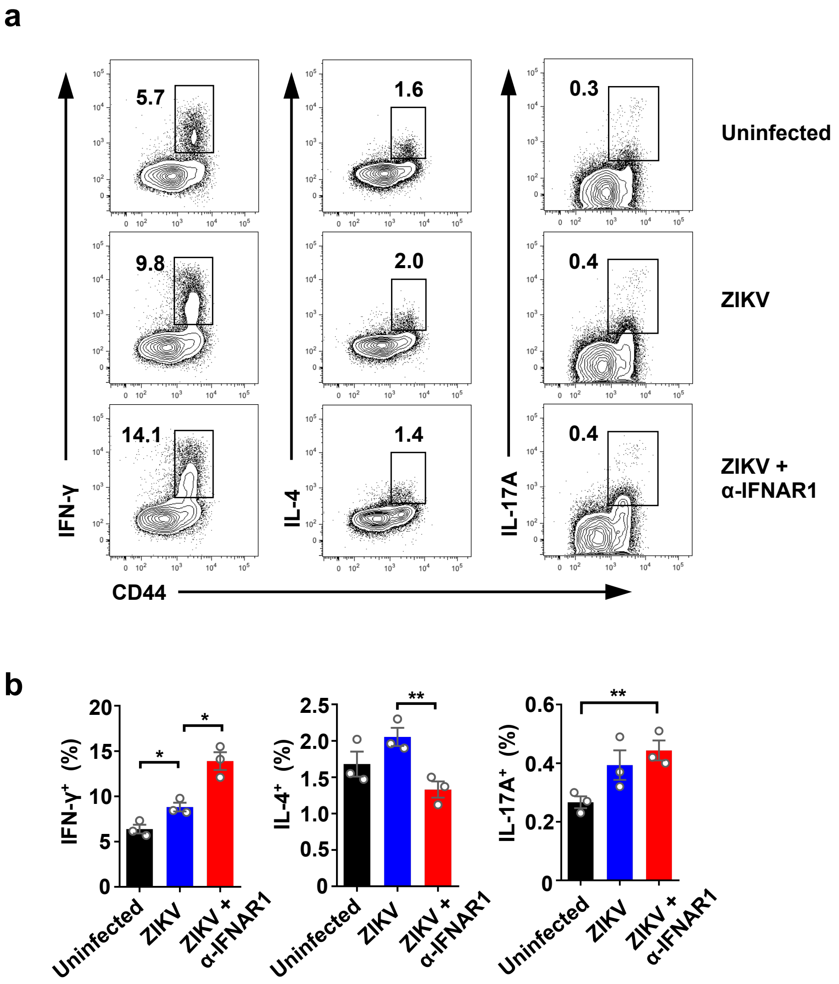

Supplementary Figure 7. CD4⁺ T cells responses after ZIKV infection.

BALB/c mice were administered with PBS, ZIKV or ZIKV with anti-IFNAR1 antibody pre-treatment. Splenocytes were collected on 7 dpi for intracellular staining. (n=3 for each group). **(a)** Representative flow cytometry plots of IFN-γ$^+$ cells (Th1), IL-4$^+$ cells (Th2) and IL-17A$^+$ (Th17) cells in CD4$^+$ T cells; **(b)** bar graphs summarized the percentages of each cell type. The summary data were presented as mean ± SEM. Statistical differences were determined by Student's t test and p values were indicated by * ($p<0.05$), or ** ($p<0.01$). Source data are provided as a Source Data file.

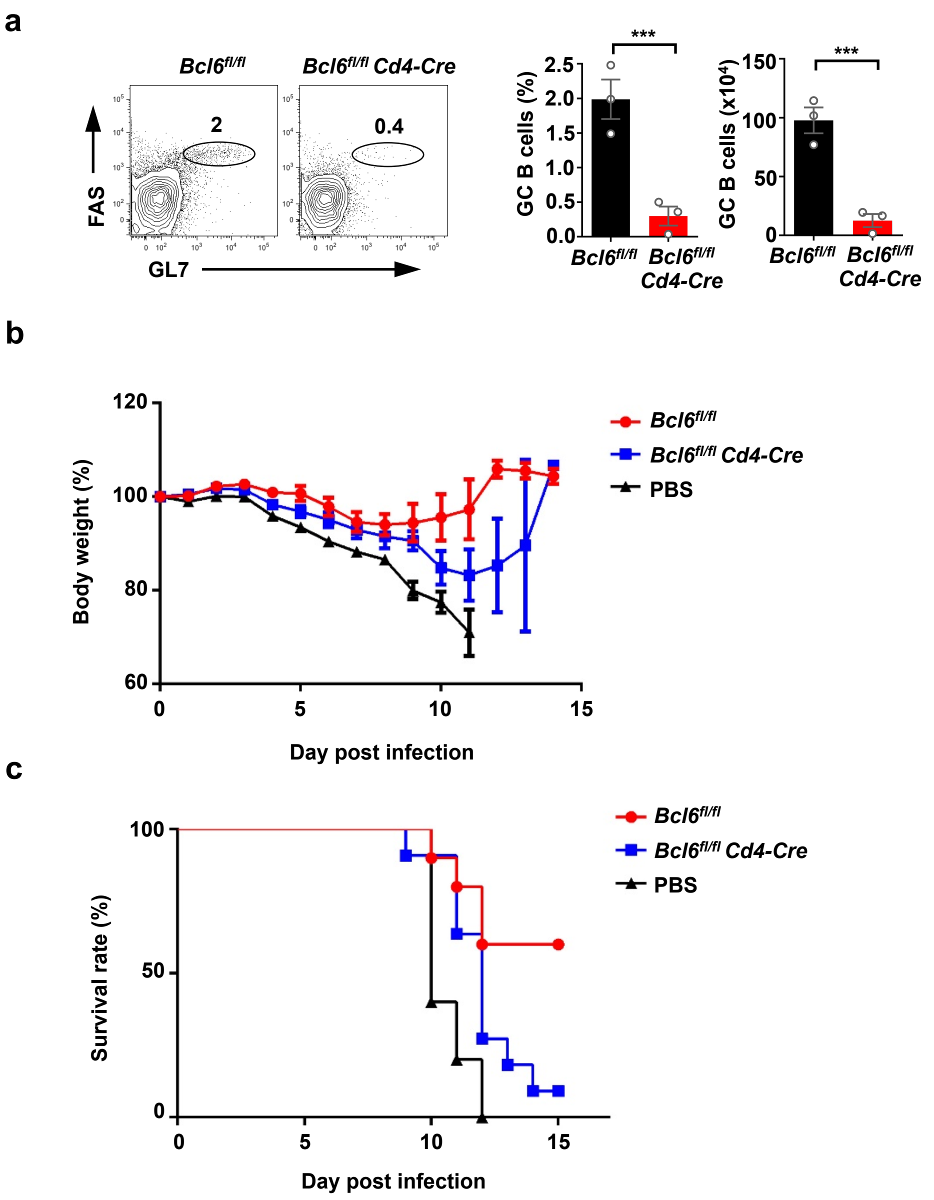

Supplementary Figure 8. Sera from *Bcl6$^{fl/fl}$Cd4*-Cre mice provide less short-term protection against ZIKV challenge than that from WT mice.

(a) Representative flow cytometry plots of FAS$^+$GL7$^+$ cells in B220$^+$ B cells (GC B cells) (Left panel); bar graphs summarized the percentages and numbers of in each group (Right panel) on 14 dpi (n=3 for each group). **(b-c)** AG6 mice were adoptively transferred with 125μl of pooled sera from PBS treated WT mice, ZIKV+ anti-IFNAR1 antibody infected *Bcl6$^{fl/f}$* (*Bcl6$^{fl/fl}$*), or ZIKV+ anti-IFNAR1 antibody infected *Bcl6$^{fl/fl}$Cd4*-Cre mice (*Bcl6$^{fl/fl}$Cd4*-Cre) on 14 dpi (n=10-11 for each group), and then challenged with ZIKV (1*10^3 PFU) one day later. The percentage of **(b)** body weight and **(c)** survival rate in each group were summarized. The summary data were presented as mean ± SEM. Statistical differences were determined by Student's t test and p values were indicated by *** (p<0.001). Source data are provided as a Source Data file.

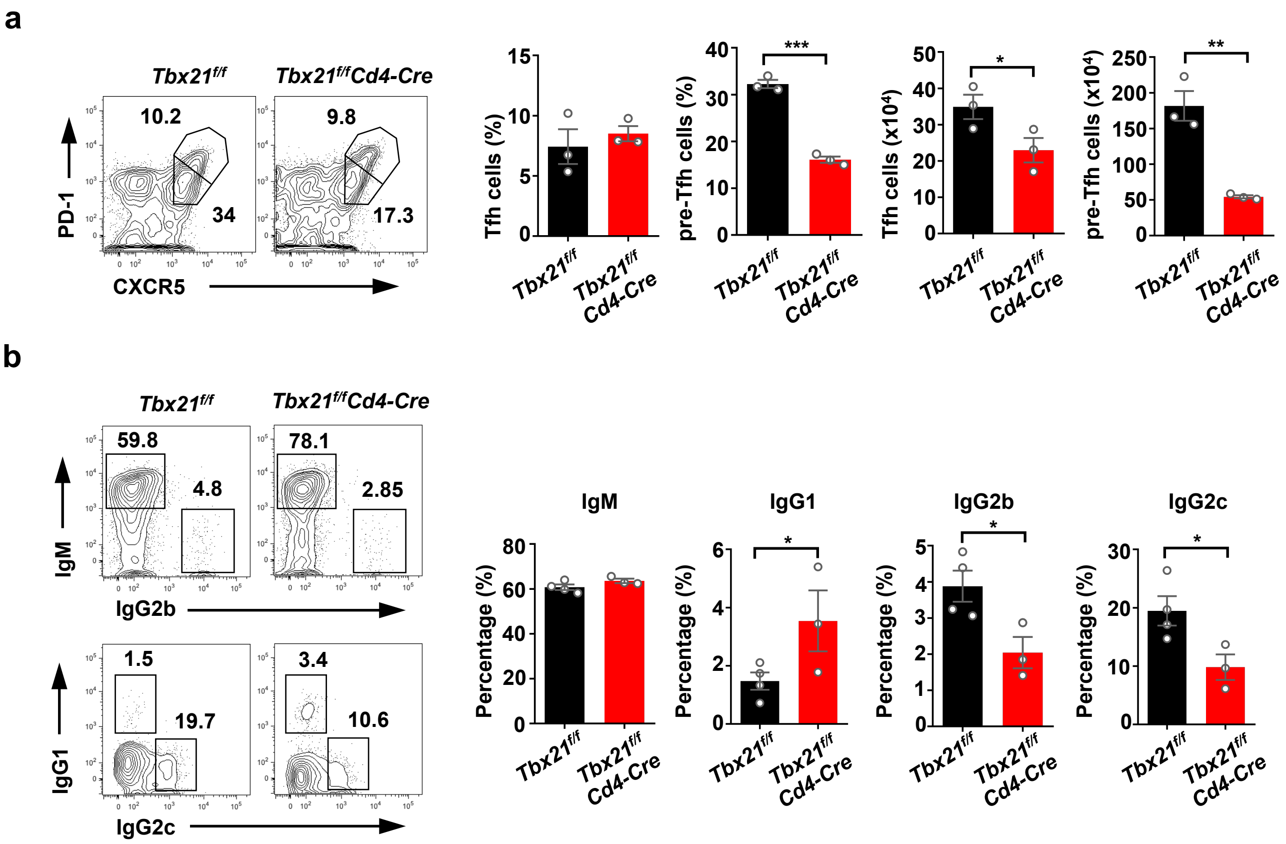

Supplementary Figure 9. T-bet is essential for Th1-like Tfh cell differentiation and IgG2c antibody class switching.

(a, b) WT mice and *Tbx21^fl/fl^Cd4*-Cre mice were infected by ZIKV with anti-IFNAR1 antibody pretreatment (n=3-4 for each group), and spleens were harvested for analysis of Tfh cell, pre-Tfh cell and B cell responses. **(a)** Representative flow cytometry plots of $CXCR5^{high}PD\text{-}1^{high}$ cells (Tfh) and $CXCR5^{medium}PD\text{-}1^{medium}$ cells (pre-Tfh) in $CD4^{+}CD44^{high}CD62L^{low}$ T cells (Left panel); bar graphs summarized the percentages and numbers of cells in group (Right panel). **(b)** Representative flow cytometry plots of IgM, IgG1, IgG2b and IgG2c staining of IgD^{low} B cells (Left panel); bar graphs summarized cell percentages (Right panel). The summary data were presented as mean ± SEM. Statistical differences were determined by Student's t test and p values were indicated by * (p<0.05), or ** (p<0.01), or *** (p<0.001). Source data are provided as a Source Data file.

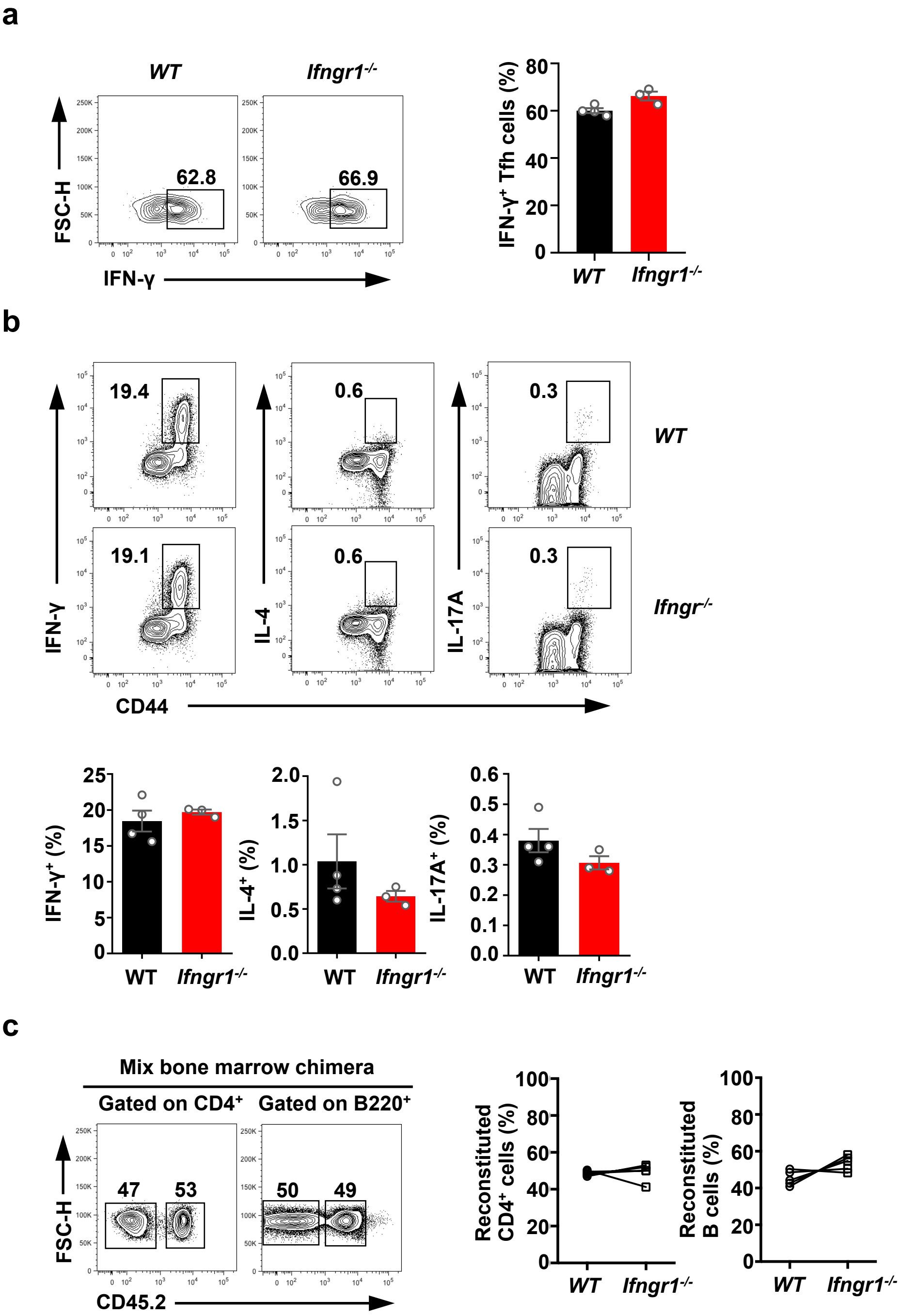

Supplementary Figure 10. The IFN-γ pathway is required for IgG2c antibody class-switching.

(a-b) WT mice, *Ifngr1$^{-/-}$* mice were infected by ZIKV after anti-IFNAR1 antibody pretreatment. splenocytes were collected on 7 dpi for detection of T cell responses (n=3-4 for each group). **(a)** Representative (upper panel) and summarized (down panel) flow cytometry data of IFN-γ production in Tfh cells. **(b)** Representative flow cytometry plots of IFN-γ^{+} cells (Th1), IL-4^{+} cells (Th2) and IL-17A^{+} cells (Th17) in CD4^{+} T cells were presented (Left panel); bar graphs summarized cell percentages (Right panel). **(c)** Representative flow cytometry analysis of CD45.2 expression in CD4^{+} T cells and B220^{+} B cells in spleen from *CD45.1^{+} WT: Ifngr1$^{-/-}$* mixed bone marrow chimeras at day 14 after ZIKV infection (Left panel); summary of the percentages of CD45.2^{-} and CD45.2^{+} CD4^{+} T cells and B220^{+} B cells (Right panel) (n=5 for each group). The summary data were presented as mean ± SEM. Statistical differences were determined by Student's t test. Source data are provided as a Source Data file.
