# Supplementary material for: ZIKV infection induces robust Th1-like Tfh cell and long-term protective antibody responses in immunocompetent mice
Source: Nat Commun. 2019 Aug 27;10:3859. doi: 10.1038/s41467-019-11754-0 (PMC6712032; doi:10.1038/s41467-019-11754-0)
Supplement: Supplementary file 3 — Reporting Summary [file 41467_2019_11754_MOESM3_ESM.pdf]

nature research

Corresponding author(s): Haikun Wang, Xia Jin

Last updated by author(s): Jul 18, 2019

Reporting Summary

Nature Research wishes to improve the reproducibility of the work that we publish. This form provides structure for consistency and transparency in reporting. For further information on Nature Research policies, see Authors & Referees and the Editorial Policy Checklist.

Statistics

For all statistical analyses, confirm that the following items are present in the figure legend, table legend, main text, or Methods section.

n/a	Confirmed	
☐	☒	The exact sample size (n) for each experimental group/condition, given as a discrete number and unit of measurement
☐	☒	A statement on whether measurements were taken from distinct samples or whether the same sample was measured repeatedly
☐	☒	The statistical test(s) used AND whether they are one- or two-sided<br>*Only common tests should be described solely by name; describe more complex techniques in the Methods section.*
☐	☒	A description of all covariates tested
☐	☒	A description of any assumptions or corrections, such as tests of normality and adjustment for multiple comparisons
☐	☒	A full description of the statistical parameters including central tendency (e.g. means) or other basic estimates (e.g. regression coefficient) AND variation (e.g. standard deviation) or associated estimates of uncertainty (e.g. confidence intervals)
☐	☒	For null hypothesis testing, the test statistic (e.g. F, t, r) with confidence intervals, effect sizes, degrees of freedom and P value noted<br>*Give P values as exact values whenever suitable.*
☒	☐	For Bayesian analysis, information on the choice of priors and Markov chain Monte Carlo settings
☒	☐	For hierarchical and complex designs, identification of the appropriate level for tests and full reporting of outcomes
☐	☒	Estimates of effect sizes (e.g. Cohen's d, Pearson's r), indicating how they were calculated

Our web collection on statistics for biologists contains articles on many of the points above.

Software and code

Policy information about availability of computer code

Data collection	Data sources are described in "Materials and Methods".
Data analysis	Graphpad version 6.0, SPSS Statistics version 19; for RNA-seq: hisat3, htseq-count, R (limma+ voom, heatmap3, Barplot), GSEA version 3.0, Flowjo 8.8.7

For manuscripts utilizing custom algorithms or software that are central to the research but not yet described in published literature, software must be made available to editors/reviewers. We strongly encourage code deposition in a community repository (e.g. GitHub). See the Nature Research guidelines for submitting code & software for further information.

Data

Policy information about availability of data

All manuscripts must include a data availability statement. This statement should provide the following information, where applicable:

- Accession codes, unique identifiers, or web links for publicly available datasets
- A list of figures that have associated raw data
- A description of any restrictions on data availability

The authors declare that the data supporting the findings of this study are available within the article and its supplementary information files, or are available upon request to the corresponding authors. The RNA-seq data that support the findings of this study have been deposited in Gene Expression Omibus with the primary accession code GSE121250. Source data are provided as a source data file.

Field-specific reporting

Please select the one below that is the best fit for your research. If you are not sure, read the appropriate sections before making your selection.

☒ Life sciences ☐ Behavioural & social sciences ☐ Ecological, evolutionary & environmental sciences

For a reference copy of the document with all sections, see nature.com/documents/nr-reporting-summary-flat.pdf

Life sciences study design

All studies must disclose on these points even when the disclosure is negative.

Sample size	For all experiments, there were minimum of 3 samples in each experiment, and data analysis showed that statistical significance was obtained from this sample size.
Data exclusions	No data was excluded in our investigation.
Replication	Replicate experiments were successfully performed.
Randomization	This is not relevant to the study and no clinical samples were involved.
Blinding	Blinding is not relevant to the study and no clinical samples were involved.

Reporting for specific materials, systems and methods

We require information from authors about some types of materials, experimental systems and methods used in many studies. Here, indicate whether each material, system or method listed is relevant to your study. If you are not sure if a list item applies to your research, read the appropriate section before selecting a response.

Materials & experimental systems

n/a	Involved in the study	
☐	☒	Antibodies
☐	☒	Eukaryotic cell lines
☒	☐	Palaeontology
☐	☒	Animals and other organisms
☒	☐	Human research participants
☒	☐	Clinical data

Methods

n/a	Involved in the study	
☒	☐	ChIP-seq
☐	☒	Flow cytometry
☒	☐	MRI-based neuroimaging

Antibodies

Antibodies used	All antibody informations were provided in Supplementary Table 1.
Validation	All antibodies were validated in previous publications.

Eukaryotic cell lines

Policy information about cell lines

Cell line source(s)	Vero E6 (ATCC, CRL-1586)<br>C 6/36 (ATCC, CRL-1660)
Authentication	Cell lines were authenticated by morphology.
Mycoplasma contamination	No contamination was observed.
Commonly misidentified lines (See ICLAC register)	N/A

Animals and other organisms

Policy information about studies involving animals; ARRIVE guidelines recommended for reporting animal research

Laboratory animals	Detailed informations were provided in "Materials and Methods, 'Mice' subsection".

Wild animals	No wild animals were involved in this study.
Field-collected samples	No field-collected samples was used in this study.
Ethics oversight	All mouse experiments were performed strictly according to roles of care and use of laboratory animals established by Institutional Animal Care and Use Committee of the Institut Pasteur of Shanghai, Chinese Academy of Sciences (Approval number: A2018027).

Note that full information on the approval of the study protocol must also be provided in the manuscript.

Flow Cytometry

Plots

Confirm that:

☐ The axis labels state the marker and fluorochrome used (e.g. CD4-FITC).

☒ The axis scales are clearly visible. Include numbers along axes only for bottom left plot of group (a 'group' is an analysis of identical markers).

☒ All plots are contour plots with outliers or pseudocolor plots.

☒ A numerical value for number of cells or percentage (with statistics) is provided.

Methodology

Sample preparation	Detailed informations were provided in "Materials and Methods, 'Mouse infection, immunization and sample collection','Construction of mixed bone marrow chimeras','Flow cytometry', 'RNA-seq analysis' subsection" and all Figure legends.
Instrument	BD Fortessa, BD Celesta and BD FACS Aria II
Software	FACSDiva for data acquisition and FlowJo 8.8.7 for post-acquisition analyzes.
Cell population abundance	the final population we showed were collected at least 1,000 events
Gating strategy	Cell debris were excluded by SSC-A and FSC-A gating (G1), Doublets were excluded by FSC-A and FSC-H gating (G2) and SSC-A and SSC-W(G3) for all flow cytometry analysis. Tfh and pre-tfh cells were gated by CD4 positive and FSC-H (G4), CD44 high and CD62L low (G5), GC B cells were gated by B220 positive and FSC-H(G4), cytokines (not in tfh) production were gated by CD4 positive and CD44 high(G4), cytokines (IFN-γ in tfh or pre-tfh) production were gated by CD4 positive and CD44 high(G4) and CXCR5 high (or medium) and PD-1 high (or medium)(G5), IgM and IgG2b were gated by B220 positive and IgDlow (G4) and IgG1 negative and IgG2c negative(G5),IgG1 and IgG2c were gated by B220 positive and IgDlow (G4) and IgM negative and IgG2b negative(G5),

☒ Tick this box to confirm that a figure exemplifying the gating strategy is provided in the Supplementary Information.
